# β-glucans: wide-spectrum immune-balancing food-supplement-based enteric (β-WIFE) vaccine adjuvant approach to COVID-19

**DOI:** 10.1080/21645515.2021.1880210

**Published:** 2021-03-02

**Authors:** Nobunao Ikewaki, Masaru Iwasaki, Gene Kurosawa, Kosagi-Sharaf Rao, Johant Lakey-Beitia, Senthilkumar Preethy, Samuel JK Abraham

**Affiliations:** a Department of Medical Life Science, Kyushu University of Health and Welfare, Nobeoka, Japan; b Institute of Immunology, Junsei Educational Institute, Nobeoka, Miyazaki, Japan; c Centre for Advancing Clinical Research (CACR), University of Yamanashi - School of Medicine, Chuo, Japan; d Department of Academic Research Support Promotion Facility, Center for Research Promotion and Support, Fujita Health University, Aichi, Japan; e Instituto de Investigaciones Científicas y Servicios de Alta Tecnología (INDICASAT AIP), City of Knowledge, Panama City, Panama; f The Fujio-Eiji Academic Terrain (FEAT), Nichi-In Centre for Regenerative Medicine (NCRM), Chennai, India; g Edogawa Evolutionary Laboratory of Science (EELS), Edogawa Hospital, Tokyo, Japan; h The Mary-Yoshio Translational Hexagon (MYTH), Nichi-In Centre for Regenerative Medicine (NCRM), Chennai, India; i Biomaterials and Cell Biology Division, JBM Inc, Tokyo, Japan; j Immune Systems R & D Division, GN Corporation Co. Ltd, Kofu, Japan

**Keywords:** Covid-19, vaccine adjuvant, AFO-202 Beta glucan, trained Immunity, immune enhancement

## Abstract

Conventional vaccines to combat COVID-19 through different approaches are at various stages of development. The complexity of COVID-19 such as the potential mutations of the virus leading to antigenic drift and the uncertainty on the duration of the immunity induced by the vaccine have hampered the efforts to control the COVID-19 pandemic. Thus, we suggest an alternative interim treatment strategy based on biological response modifier glucans such as the *Aureobasidium pullulans* AFO-202-derived β-glucan, which has been reported to induce trained immunity, akin to that induced by the Bacille Calmette-Guérin vaccine, by epigenetic modifications at the central level in the bone marrow. These β-glucans act as pathogen-associated molecular patterns, activating mucosal immunity by binding with specific pathogen recognition receptors such as dectin-1 and inducing both the adaptive and innate immunity by reaching distant lymphoid organs. β-Glucans have also been used as immune adjuvants for vaccines such as the influenza vaccine. Therefore, until a conventional vaccine is widely available, an orally consumable vaccine adjuvant that acts like biosimilars, termed as the wide-spectrum immune-balancing food-supplement-based enteric (β-WIFE) vaccine adjuvant approach, with well-reported safety is worth in-depth investigation and can be considered for a clinical trial.

## Background

The COVID-19 pandemic is wreaking havoc on the lives of billions of people worldwide, with unprecedented consequences and implications. COVID-19 biology and pathology are very complex, posing a big challenge in clinical and drug management. Hence, researchers across the globe are creating strategies for developing drugs, antibodies, vaccines, and other therapies to fight the causative virus, SARS-CoV-2.^1^ According to the World Health Organization (WHO) [last accessed December 19, 2020], there are 202 companies and universities worldwide working on a SARS-CoV-2 vaccine.^2^ The vaccines mRNA-1273 (Moderna), Ad5-nCoV (CanSino Biologicals), INO-4800 (Inovio, Inc.), LV-SMENP-DC, Pathogen-specific aAPC (ShinzenGeno-Immune Medical Institute), and ChAdOx1 (University of Oxford) have entered the phase I/II clinical trials.^2,3^ Authorisation/approval has been obtained in some countries such as China, United Arab Emirates, and Russia for six vaccines: CoronaVac by Sinova in China; Inactivated vaccine by Wuhan Institute of Biological Products in China and National Pharmaceutical Group (Sinopharm) in China, BBIBP-CorV by Sinopharm in China, United Arab Emirates, and Bahrain; Sputnik V, Non-replicating viral vector by Gamaleya Research Institute, Acellena Contract Drug Research and Development in Russia; EpiVacCorona, Peptide vaccine by Federal Budgetary Research Institution State Research Center of Virology and Biotechnology in Russia; and BNT162b2, mRNA-based vaccine by Pfizer, BioNTech, and Fosun Pharma in UK, Bahrain, Canada, Mexico, USA, Singapore, Oman, Saudi Arabia, and Kuwait.^4^ As of July 2020, there were over 158 vaccine candidates, 135 of which were in the preclinical or exploratory stage of development,^5^ virtually all concentrating on inducing neutralizing antibodies (nAbs) against the spike (S) protein on the virus surface.^1,6^

Vaccine approaches conventionally use live attenuated viruses, inactivated virus proteins, polysaccharide-conjugated subunits, virus-like particles, nucleic acid (DNA and RNA), viral vectors, and recombinant proteins. The vaccine’s ability to induce cellular immunity (other than B-cell-produced antibodies) has been indicated as necessary for a rational vaccine design, because nAb responses wane rapidly.^1^ Furthermore, the coronavirus genome is highly prone to mutations that can lead to genetic drift and escape immune recognition. In fact, several variants that can cause drifts have already been identified.^7^ Undesired immunopotentiation in the form of eosinophilic infiltration or increased infectivity has limited the exploration of some of the COVID-19 vaccine candidates and is currently a challenge in vaccine biology.^7^

An ideal vaccine should meet all or most of the following criteria:

- It should offer wide-spectrum protection across various substrains and novel variants that are emerging or may emerge later
- It should possess characteristics such as minimal undesired immunopotentiation
- It should be suitable for stockpiling for adult healthcare workers and for adults > 60 years old or for whom has underlying diabetes or hypertension^8^
- It should induce long-lasting effective immunity in all vaccinated subjects across ages
- It should be safe, stable, and easily available and administrable

Given the above criteria, we evaluated the suitability of β-glucans as they have been reported to exert several beneficial effects on human and animal health.^9^

## β-glucans and immunity

Trained immunity (TRIM) induction is a promising defense strategy against COVID-19.^10^ The widely known Bacille Calmette-Guérin (BCG) vaccine induces TRIM, protects against severe forms of tuberculosis (TB) caused by *Mycobacterium tuberculosis*, with limited effect against pulmonary tuberculosis, and confers nonspecific protective properties against unrelated infections and mortality.^10^ BCG’s nonspecific protection is T-cell and B-cell independent and is mediated by the functional and epigenetic reprogramming of innate immune cells such as monocytes, macrophages, and natural killer (NK) cells. This protection is called TRIM. TRIM has been reported to be induced by stimulants like β-glucans, LPS, or the BCG vaccine.^11^ We focused on how β-glucans could serve as inducers of TRIM in the context of a wide-spectrum vaccine adjuvant approach to COVID-19.

## β-glucans and TRIM

β-Glucans are a heterogeneous group of polysaccharides abundant in the cell walls of yeasts, bacteria, and fungi that reportedly induce TRIM. β-Glucans induce epigenetic reprogramming in innate immune cells, leading to cellular activation, augmented cytokine production, and changes in metabolic function that shift cellular metabolism from oxidative phosphorylation to glucose fermentation mediated by the Akt/mammalian target of rapamycin (mTOR)/hypoxia-inducible factor 1α (HIF1α) pathway,^12^ thus effectively inducing TRIM. Epigenetic alterations such as histone methylation and acetylation lead to the positive regulation of gene expression. When such epigenetically “trained” cells have contact with heterologous secondary stimuli, they are programmed to produce a more robust immune response.^10,11^ The cells are reportedly not peripherally trained, but β-glucans can impact the bone marrow (BM) and lead to a lasting TRIM phenotype.

## Arms of the immunity stimulated by β-glucans and implications in COVID-19

Administering intraperitoneal β-glucans specifically expand Lin−Sca1+ cKit+ (LSKs) and multipotent myeloid progenitor 3 (MPP3)-expressing hematopoietic stem cells (HSC) in the BM, and such trained HSCs generate a “central” memory.^12,13^ Epigenetic modifications driven by β-glucans are rapidly activated by secondary infections or stimuli such as viruses and may serve as a potent strategy for vaccines against COVID-19.^8^ β-Glucans act as pathogen-associated molecular patterns (PAMPs), because they are present in the cell wall of several pathogenic yeasts and bacteria and are involved in microorganism recognition and clearance by the human immune system. Upon reaching the intestine, β-glucans are internalized by intestinal epithelial and/or M cells and presented to immune cells within the Peyer’s patches. β-Glucan particles can also reach distant lymphoid organs via the blood or lymph. In the Peyer’s patches, β-glucan particles are recognized by the ligation of specific pathogen recognition receptors (PRRs) such as toll-like (TLR) and C-type lectin-like receptors. Among C-type lectin-like receptors, dectin-1 is the most-studied receptor that binds β-glucan from various sources. Dectin-1 is expressed on the surface of monocytes, macrophages, neutrophils, dendritic cells, and T lymphocytes, which are all activated by β-glucan binding. This binding leads to a number of cellular responses via the modulation of inflammasome and transcription factor activation, which results in the production of cytokines, chemokines, and reactive oxygen species. β-Glucans stimulate NK cell cytotoxic activity as part of the innate immune response by binding directly to the NKp30 activating receptor.^13,14^ β-Glucan innate immunity targets are monocytes, macrophages, dendritic cells, and NK cells. β-Glucans induce the antimicrobial activity of mononuclear cells and neutrophils as well.^13^ Regarding T cells, β-glucans help in CD4 + T-cell immunomodulation, allowing them to infiltrate tumors and thereby inhibit tumor growth.^15^ Orally administered β-glucans can reach the spleen and lymph nodes and significantly reduce tumor burdens by activating DCs, expanding and activating antigen-specific CD4 and CD8 T cells, and inducing IFN-γ production.^16^ β-Glucans also induce B lymphocytes to produce antibodies. Short-term supplementation with β-glucans improves the levels of salivary immunoglobulins (sIgM, sIgG, and sIgA).^16^ Orally administered β-glucans significantly stabilize IgG1 levels, maintaining anti-infectious immunity. Thus, all aspects of the immune system are activated and modulated by β-glucans, making them worth considering as an ideal vaccine that produces long-lasting effective immunity, is broadly protective, is effective in all vaccinated subjects across ages, and is stable and easily administrable.^17,18^

## β-glucan supplementation for COVID-19

Long-lasting immunity is currently a big challenge in COVID-19-affected patients.^17^ β-Glucans can produce long-lasting TRIM against a wide range of pathogens.^18^ Furthermore, β-glucans are safe for consumption at all ages, and they fall under the FDA’s generally recognized as safe category.^19^ β-Glucans are stable and can be consumed continuously as a food supplement.^9^ Many types of β-glucans exist, but yeast- and mushroom-derived β-glucans exert stronger immunomodulatory effects than do other types of β-glucans.^20^ Oral β-glucans have been thoroughly described as prophylactic supplements to boost immune responses and to abrogate COVID-19 symptoms via their TRIM actions.^10^ Though SARS-CoV-2 is predominantly considered a virus that affects the respiratory system, the viral host receptor ACE2 appears in the cytoplasm of gastrointestinal epithelial cells, with the viral nucleocapsid protein appearing in the cytoplasm of rectal, duodenal, and gastric epithelial cells, suggesting that the intestine may be relevant in the pathogenesis of COVID-19 and maybe a possible route of infection.^21^ β-Glucans with immune effects on the intestine may therefore be an advantageous supplementation strategy for COVID-19 therapy. Gut-dysbiosis is also a key element in determining infection-related diseases. β-Glucans can also modulate the gut bacteria, improving immune response.^22^ β-Glucan supplements decrease the incidence of upper respiratory tract infections in randomized control trials.^23,24,25,26–^^27^ A β-glucan extract from the edible shiitake mushroom *Lentinus edodes* has recently been reported to show differential *in vitro* immunomodulatory and pulmonary cytoprotective effects and may be indicated for COVID-19 immunotherapy.^27^ The study compared two types of *Lentinan* extracts that differentially reduced cytokine-induced NF-κB activation in human alveolar epithelial A549 cells and attenuated pro-inflammatory cytokine production (TNF-α, IL-8, IL-2, IL-6, and IL-26), as well as TGF-β and IL-10 secretion. The study suggested that β-glucans delivered as a tailored cocktail might fit future nutraceutical-based intervention for COVID-19. The study also mentioned a major drawback: maintaining functional bioactivity and increasing the β-glucan yield required less harmful extraction processes without enzyme and harsh chemical usage.^27^ This extraction process is crucial for using β-glucans against COVID-19.^28^

## AFO-202 β-glucan

We herein describe the β-glucan derived from the black yeast *Aureobasidium pullulans* AFO-202 strain that is uniquely secreted as an exopolysaccharide. Therefore, it does not need any kind of extraction-to-purification procedure, resulting in highly pure β-glucan with significant bio-functional activity.^28^ The AFO-202 β-glucan can induce various positive immune responses relevant to COVID-19. It decreased levels of IL-6, which is commonly the most elevated cytokine in a COVID-19 cytokine storm, the main mechanism leading to organ damage and mortality. It enhances IFN-γ and sFAS levels. It is associated with increased production of IL8, which causes the activation, migration, and chemotaxis of cytotoxic neutrophils. It decreases CCL2 and CXCL10 levels, inhibiting the chemoattraction of monocytes/macrophages, T cells, NK cells, and dendritic cells. The prevention of chemoattraction modulates immune responses. It also increased IL-7 production, leading to mature T-cell survival and development. Activating CD8+ (cytotoxic T cells), CD4+ (mainly Th1 cells), and Treg cells helps balance the regulatory immune response. Activation of B cells by AFO-202 β-glucan results in the production of virus-specific antibodies.^28,29^ AFO-202 β-glucan enhances NK cell activity against *Leishmania amazonensis* infection.^30^ AFO-202 β-glucan is also present in the inner wall of *Candida albicans*, making it a strong PAMP candidate with a significant chance of recognition by PRRs. AFO-202 β-glucan has been consumed since 1996, when the Japanese Regulatory Authority approved it as a food additive. It has been established as safe and efficacious in elderly patients.^31^ AFO-202 β-glucan also helps to maintain blood glucose and lipid levels,^32,33^ addressing the high risk of comorbidities like diabetes and cardiac diseases in the pathogenesis of COVID-19. As fasting plasma glucose has been indicated as an independent predictor of the outcome at the time of admission in COVID-19 patients, the metabolic effects of AFO-202 β-glucan may have prophylactic potential in COVID-19.^34^ In addition, AFO-202 β-glucan has been suggested to be beneficial in decreasing the risk of coagulopathy due to the presence of a dysregulated inflammatory system in COVID-19 patients, especially in vulnerable individuals based on race (Caucasians, African Americans and Hispanics), people with comorbidities, including diabetes, hypertension, and cardiovascular diseases, pregnant women.^35^

## AFO 202 β-glucan as a wide-spectrum immune effector

Oral vaccines generate immunity in gut-associated lymphoid tissue (GALT) that consists of lymph nodes, Peyer’s patches (containing 75% of B cells and 20% of T cells), and isolated lymphoid follicles in the gastrointestinal tract (GIT).^36^ M cells transport the antigen in the vaccine across the mucosal barrier into Peyer’s patches, and the antigen is presented to T cells by antigen-presenting cells. CD4 + T cells are activated, supporting the germinal center development, B-cell affinity maturation, and class-switching to IgA, along with CD40/CD40 ligand interactions and cytokine secretion. The antigen-primed B cells migrate to distant effector regions, where they differentiate into plasma cells that secrete dimeric or polymeric IgA molecules. These molecules are transported into the intestinal lumen as secretory IgA (sIgA) that can prevent attachment and pathogen invasion, neutralize enterotoxins, and induce serum IgG via the vaccine, acting against mucosally and systemically invasive pathogens. Vaccines also activate cell-mediated immune responses against intracellular bacteria and viruses, along with antibody-dependent cellular cytotoxicity responses.^37,38^

Following intra-dermal vaccination, immune cells such as DCs, T lymphocytes, NK cells, macrophages, and mast cells present in the skin epithelium trigger the skin’s inflammation cascade, mainly via Langerhans cells (a specific DC subset that migrates into the lymph nodes following antigen capture and initiates the adaptive immune response). These cells are stimulated by PAMPs via an array of germline-encoded PRRs, including TLR and langerin (CD207). The skin’s resident mast cells induce the innate immune response in the skin by releasing granules containing inflammatory mediators.^39^

The immune system triggers pathways of oral and intradermal vaccination depend on components of the reticuloendothelial system or the mononuclear phagocyte system^39^ employed to access the immune system. Oral vaccines start with mucosal-associated lymphoid tissue and GALT, while intra-dermal vaccines start with peripheral lymphoid tissues.

## AFO-202 β-glucan as a vaccine adjuvant

β-Glucans have been suggested to be promising anti-infective vaccine adjuvants, as they alone can stimulate various immune reactions, including antibody production without any adverse reactions. β-Glucans have been employed as adjuvants to vaccines against *Yersinia ruckeri*.^39^ β-Glucans as adjuvants have been found to enhance immunogenicity of hepatitis B vaccine, influenza vaccine, and vaccines against systemic aspergillosis and coccidioidomycosis. The AFO-202 β-glucan has been proven to be a potential immune adjuvant, because when it was administered with an avian influenza H5 subtype vaccine, it elicited strong immune responses with high hemagglutination inhibition titers and 10–20% ELISA seroconversion.^40^

**Figure 1.**
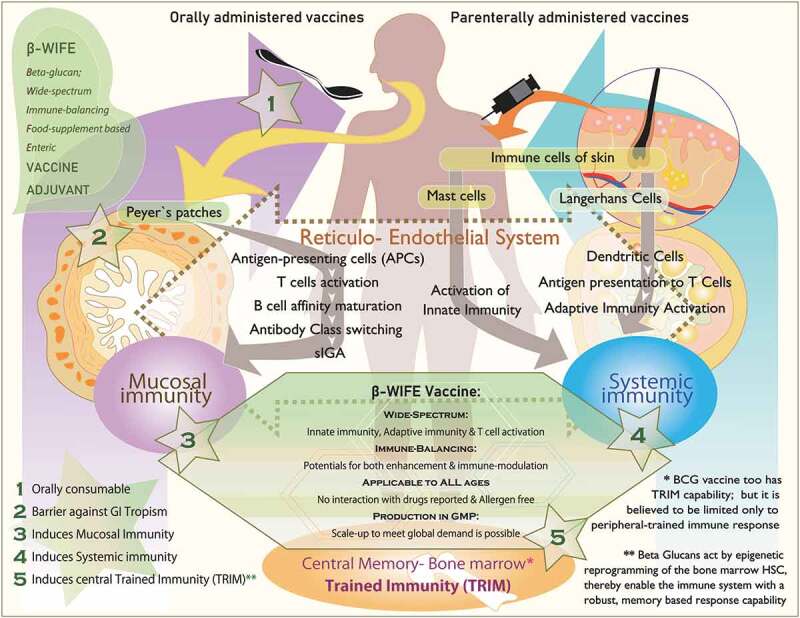
Schematic illustration describing (i) stepwise, the mechanisms of orally administered vaccines starting from Peyer’s patches of the gut to induce mucosal immunity and parenteral vaccines starting from immune cells of the skin to induce systemic immunity, (ii) ability of β-glucans to activate all aspects of the immune system including the central trained immunity (TRIM) of bone marrow and (iii) the strategic key advantages of β-glucans at five different stages and actions to play a role as vaccine adjuvant

Figure 1 illustrates the mechanisms and pathways that differ between oral and intra-dermal vaccines, and how β-glucans interact with components involved in both the types of vaccines. Table 1 provides the details on the various immune effector mechanisms and pathways through which β-glucans may be able to serve as vaccine adjuvants for COVID-19 therapy.

**Table 1.** Various immune effector mechanisms and pathways through which β-glucans may be able to serve as vaccine adjuvants for COVID-19 therapy

S. No	Immune mechanisms of β-glucans as vaccine adjuvants for COVID-19 therapy	Reference(s)
1	Trained immunity (TRIM); both central and peripheral	10–12
2	Epigenetic reprogramming in innate immune cells	11
3	Activation of pathogen recognition receptors (PRRs) such as toll-like (TLR) and C-type lectin-like receptors, including dectin-1	12,13
4	Stimulation of monocytes, macrophages, dendritic cells, and NK cells	13,14
5	Enhancing antimicrobial activity of mononuclear cells and neutrophils	13,14
6	Expanding and activating antigen-specific CD4 and CD8 T cells	15,16
7	Antiviral response by stimulating production of IFN-γ	15
8	Induction of B lymphocytes to produce antibodies	16
9	Increasing of salivary immunoglobulins (sIgM, sIgG, and sIgA) for saliva-based mucosal immunity	16
10	Induction of mucosal immunity including that of the gut by acting on reticuloendothelial system	8,13,39,41
11	Immune balance against cytokine storm by decreasing levels of IL-6, CCL2, and CXCL10 levels and increasing levels of IL-7, IL-8, IFN-γ, and sFAS	28,35,36

## β-glucans as wide-spectrum immune-balancing food-supplement-based enteric (β-WIFE) vaccine adjuvant approach to COVID-19

β-Glucans induce TRIM with epigenetic reprogramming in innate immune cells at the BM level, leading to a long-lasting central and peripheral TRIM.^9–11^ Since β-glucans are recognized as PAMPs, they are recognized by the ligation of specific PRRs, such as TLR and C-type lectin-like receptors, which stimulate both innate immunity by targeting cells, including macrophages and NK cells, as well as adaptive immunity by expanding and activating antigen-specific CD4 and CD8 T cells and enabling B lymphocytes to produce antibodies.^15,16,28^ β-Glucans also enhance mucosal immunity, employing the majority of the components of the reticuloendothelial system by inducing gut mucosal immunity, traveling to distant effector sites such as the spleen and lymph nodes.^39,41^ β-Glucans activate all aspects of the immune system,^9–13^ resulting in a continuous, lasting immune response against various pathogens that can elicit specific antiviral immunity.^28^ Above all, this β-glucan-based immune response is obtained through a simple oral food supplement administration, with a proven track record of safe consumption at all ages,^28^ besides having been employed as vaccine adjuvants.^37,38,40^ Thus, for COVID-19, we have termed this approach as a β-WIFE vaccine adjuvant approach.

Our group has recently initiated a pilot study in healthy volunteers (men aged between 40 and 60) on evaluation of biomarkers relevant to thrombogenicity, apart from immune enhancement and immune modulation with AFO-202 β-glucan and the interim results are encouraging. Based on these encouraging preliminary results, we plan to undertake a controlled study in COVID-19 patients whose general health conditions permit oral consumption.

## Conclusion

Without definitive therapeutics for COVID-19, and although some vaccine candidates have been authorised/approved, significant hurdles still exist in identifying an ideal vaccine with a wide-spectrum activity and no side effects. Orally consumed β-glucans such as the AFO-202 β-glucan might be able to serve as a β-WIFE vaccine adjuvant approach to COVID-19. However, this approach needs extensive validation by multi-centric randomized clinical trials.
